# Application of Voronoi Tessellation to the Additive Manufacturing of Thermal Barriers of Irregular Porous Materials—Experimental Determination of Thermal Properties

**DOI:** 10.3390/ma18081873

**Published:** 2025-04-19

**Authors:** Beata Anwajler

**Affiliations:** Department of Energy Conversion Engineering, Faculty of Mechanical and Power Engineering, Wroclaw University of Science and Technology, 27 Wybrzeze Wyspianskiego Street, 50-370 Wroclaw, Poland; beata.anwajler@pwr.edu.pl

**Keywords:** thermal insulation, additive manufacturing, 3D printing, Voronoi cells, thermal properties, biodegradation

## Abstract

The issue of energy transfer is extremely important. In order to achieve the lowest possible energy consumption and the required thermal efficiency in energy-efficient buildings, it is necessary, among other things, to minimize the heat-transfer coefficient, which depends on the properties of the insulating material. Analyses of the relationship between the structure of a material and its thermal conductivity coefficient have shown that lower values of this coefficient can be achieved with a more complex structure that mimics natural forms. This paper presents a design method based on the Voronoi diagram to obtain a three-dimensional structure of a porous composite material. The method was found to be effective in producing structures with predefined and functionally graded porosity. The porous specimens were fabricated from a biodegradable soybean oil-based resin using mSLA additive technology. Analyses were performed to determine the thermal parameters of the anisotropic composites. Experimental results showed that both porosity and irregularity affect the thermal properties. The lowest thermal conductivity coefficients were obtained for a 100 mm-thick prototype composite with the following parameters: wall thickness *D* = 0.2 mm, cell size *S* = 4 mm, number of structural layers *n* = 2, and degree of irregularity *R* = 4. The lowest possible thermal conductivity of the insulation was 0.026 W/(m·K), and the highest possible thermal resistance was 3.92 (m^2^·K)/W. The method presented in this study provides an effective solution for nature-inspired design and topological optimization of porous structures.

## 1. Introduction

The development of modern, sustainable insulation materials should consider their environmental impact and the use of raw materials and materials from which they are made. The production of such materials is linked to environmental protection, reducing the greenhouse effect, and saving energy. Reusable and recyclable building and packaging materials are available, such as plastic products based on recycled polymers and metals [1]. The production process should use as little energy as possible, produce as little waste as possible after production, or provide waste for reuse [2]. These conditions can be met using incremental manufacturing (AM) techniques, which have many environmental advantages over conventional manufacturing methods. They result in less material loss, and manufacturing processes have lower energy consumption and greenhouse gas emissions [3]. Of the various AM-related technologies, Selective Laser Sintering (SLS), Selective Laser Melting (SLM), and Fused Deposition Modeling (FDM) are the most widely employed [4]. First, a 3D model is created using computer-aided design (CAD) software, such as SolidWorks, CATIA, Creo, or Fusion 360, or from scans obtained using imaging techniques. This model is then sliced into flat (2D) images using slicing software that works with a 3D printer. The 2D images guide the printer as it builds subsequent layers of the 3D model [5]. At the industrial level, technology has now evolved in several directions, with advanced materials, better-quality materials, larger workspaces, and new additive technologies, including 3D concrete printing (3DCP). Today, 3D printing research is also moving towards the production of materials not only from pure polymers but also from their composites [5,6]. Bioplastics, especially biodegradable and compostable ones, have emerged as an alternative for human development. The possibility of using thermoplastic waste materials through the use of 3D printing, creating innovative recycled and naturally derived materials in 3D printing, is rapidly developing to reduce environmental problems [7,8]. This is largely due to the fact that the materials produced are environmentally friendly, widely available, and cost-effective. Reinforcing plastics with biofibers or blending them with other biodegradable products has proven to be an effective way to reduce the cost and thermo-mechanical properties of some commonly used polymers to produce a fully biodegradable composite. Composites made from them have a lower environmental impact and are more biodegradable. They are, therefore, considered a “green option” for 3D printing [9,10].

The capabilities of additive manufacturing, or 3D printing, make it possible to achieve complex geometries that would be difficult to achieve with conventional construction techniques [11,12]. By controlling the internal geometry and the combination of filling percentages, 3D printing allows both the optimization of thermal conductivity and the creation of lighter components, as shown by experiments conducted by various researchers [13,14,15,16,17]. Porous materials are an important part of the global economy. They are used in many sectors, such as construction, biotechnology, automotive, aerospace, and energy. They are used in the manufacturing of sporting goods, leisure products, and various types of packaging. The structure of cellular and porous bodies is the subject of continuous research by scientists and engineers. Modern analytical techniques allow a better understanding of their properties. The range of materials from which they can be made is also constantly expanding, opening up new possibilities for their use [18]. Modification of the cell architecture offers unlimited possibilities to achieve the desired material properties [19]. Equivalent geometric models, such as metal models with a series of homogeneous cells, are often used to represent real foam structures [20]. These models, the Lord Kelvin model [21,22,23], the Weaire–Phelan model [23], the Gibson–Ashby model [24], and the Suleiman model [25], have been used to simulate heat transfer in porous foams. They show good agreement with experimental data. The Kelvin and Weaire–Phelan models are the most commonly used in numerical studies because their geometry accurately reflects the actual structure of foams [21,22,23,26].

In addition to cellular materials, there are also fibrous materials, an example of which is PMFSS [27,28]. PMFSS is a nonwoven fibrous mat composed of curved and partially overlapping sintered fibers. Various modeling techniques have been used to characterize such a porous structure and determine its thermal properties. Sadeghi et al. [29] developed a compact analytical model to evaluate the effective thermal conductivity of a fibrous gas diffusion layer (GDL). Their geometric model was an idealization of a real material consisting of uniformly spaced cylindrical fibers. Zamel et al. [30] examined digitally based stochastic models of carbon paper. The fibers were assumed to be cylindrical and arbitrarily overlapping, and the orientation of the fibers was determined using a single-parametric directional distribution to ensure that the fibers were isotropic in the in-plane direction and anisotropic in the through-plane direction. Qiu et al. [31] used μCT-based finite element analysis (FEA) to evaluate the effect of internal defects on the thermal overpotential of fiber-reinforced polymer (FRP) composites. The μCT-based modeling provides insight into the actual three-dimensional (3D) mesoscale structures for different types of defects (voids, pinholes, air pockets, and delaminations) in FRPs.

Cellular and fiber composites are widely used in the aerospace, marine, automotive, and construction industries. Thanks to 3D printing technology, they are taking on a new meaning. Lightweight sandwich panels are now widely used because of their high stiffness-to-weight ratio, excellent thermal insulation, and high energy-absorption capacity [32]. Three-dimensional printing is also a promising new technology in construction. Therefore, it may be interesting to integrate the advantages of 3D printing with those of low-carbon-footprint insulation materials. The range of potential applications for 3D-printed insulation structures is broad. One promising replacement for traditional plastics is the use of biopolymers, the global demand and production of which is expected to increase in the coming years [33].

The purpose of this study was to experimentally determine the dependence of the effect of the anisotropy of the structure of cellular composites produced by additive SLA technology from biodegradable oil-based resin on their thermal properties. In this study, a method based on Voronoi tessellation was used to construct controlled porous regular and irregular shapes. The irregularity factor in this method can provide a good fit and harmony between “irregularity” and “controllability”. The method presented in this paper provides an effective solution for nature-inspired design and topological optimization of porous structures. The method is shown to be effective in producing structures with predetermined and functionally graded porosity.

### 1.1. Parametric Design

Computer-aided design CAD/CAM/CAE now provides tools that are radically changing the practice of materials manufacturing. In general, parametric design for materials engineering allows for a faster, more efficient, and more accurate creation of new materials tailored to specific applications [34]. It is an approach in which the properties and structure of materials are defined by a set of parameters and mathematical algorithms. This allows materials to be optimized and adapted to specific applications by dynamically modifying their properties, such as chemical composition, microstructure, mechanical properties, or thermal conductivity.

The parametric approach allows control of the microstructure of materials, e.g., grain size, porosity, and phase distribution in composites. Examples include metal alloys with optimised strength and conductivity [35] or biocompatible materials with tailored porosity for implants [36]. Another important aspect is the optimization of mechanical and physical properties, as well as the design of materials with specific properties, such as lightweight but high-strength structures in the aerospace industry [37]. Another example is the use of algorithms to find the best combination of chemical composition and heat treatment [38]. The parametric approach allows the generation and testing of different configurations and also the design of optimised structures for 3D printing technology, e.g., lightweight but strong Kraft structures [39], by adjusting the geometry and composition of metallic or post-limer powders, depending on the application.

### 1.2. Design of Cellular Composites That Have a Complex Core Geometry–Voronoi Diagram

One of the algorithms widely used in graphics, industrial design, and architecture to generate visually appealing patterns is plane tessellation using a Voronoi diagram [40,41]. The high popularity of the use of the diagram can be explained by the legitimate association of the pattern with forms created by nature, e.g., the structure of a leaf.

Voronoi tessellation is a term from the early 20th century, named after the Russian algebraist and mathematician Georgy Fedoseevich Voronoi (1868–1908). It refers to a basic geometric construction based on a particular set of points [42,43]. It allows space to be divided into polygons of different shapes, which is useful in designing optimal material structures [43]. Methods for generating these subdivisions include numerical and optimization algorithms such as the Monte Carlo method and algorithms based on Delaunay triangulation. Their properties, such as anisotropy, porosity control, and adaptability, make them attractive in fields as diverse as architectural design, fluid flow analysis, and modelling of biological structures.

The Voronoi diagram is commonly used to approximate and pattern a variety of cellular systems and random patterns found in nature. It allows the characterization of materials with cellular structures such as sponge bone, multicrystalline alloys, or foams [44]. Tessellation is often used to generate zones of facades, internal partitions, the arrangement of lighting panels, or suspended ceilings. The Voronoi Diagram can also be applied to three-dimensional models, as it offers extensive possibilities for technological innovation due to the effective partitioning of space into 3D zones. In the field of engineering, for example, Voronoi partitioning can be used to create structural concepts. This includes the design of prostheses that mimic the intrinsic cellular properties of human bone for biomedical purposes by adjusting the internal density of models [45,46,47] or in structures for industrial applications such as the manufacture of tires with internal porosity [48,49].

Voronoi cell-based porous structures are generally divided into regular and irregular [19]. The cell unit method and the triple periodic minimum surface method are known as the basic ways to construct regular porous structures. Regular porous forms are characterized by regular pore morphology, good connectivity of cell-forming supports, and easy modeling and control of thermal and mechanical properties. In contrast, irregular porous forms are usually implemented using mathematical models and computer programs. Uncontrolled irregularity leads to poor reproducibility of morphology and physical properties. Therefore, it is desirable that irregular porous forms have precisely designed geometric parameters and are distributed in a gradient manner. The approach to designing an irregular porous structure is another key technology that requires further research. As a result, irregular porous scaffolds will find greater application in the simulation of complex and anisotropic microstructures, providing additional freedom for bionic design and topological optimization and offering larger surface area and better connectivity compared to regular or uniform structures [50,51]. Such a solution allows full control of the structures and their use in a variety of fields.

This paper presents a parametric design method for porous structures that defines a functional relationship between porosity (P) and ray radius (R) based on a three-dimensional graph and an attractor. This design method allows not only the creation of lattice structures with uniform or graded porosity but also the creation of customized lattice structures tailored to the porosity of individual unit cells.

## 2. Materials and Methods

The methodology and implementation of the research will involve conducting an experiment to determine the thermal conductivity coefficient and thermal resistance of composite materials, considering their variable porosity, the gradient of irregularity in the distribution and thickness of the surface cell walls, and the multilayer structure of the composites. There are many methods for measuring the thermal conductivity of materials. The method chosen for this study is that described in ISO 9869-1:2014 [52].

### 2.1. Materials: Irregular Porous Structure Design

**Figure 1:**
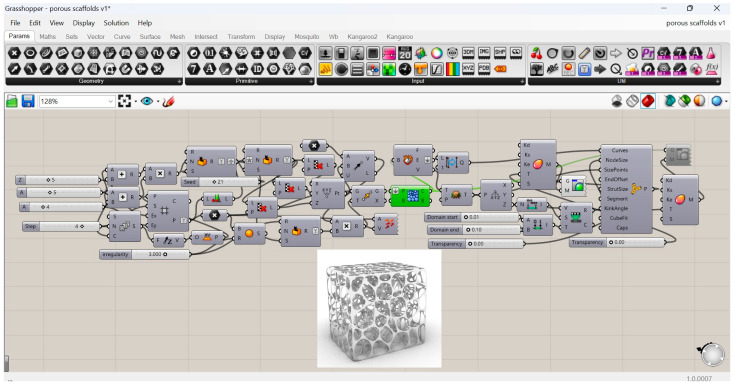
An exemplary program created in Rhino 7 for designing cores with complex geometrical parameters in cellular composites [own elaboration].

In the study, a top-down design method based on the Voronoi tessellation method was used to generate cellular composites. A programme was used to generate regular and anisotropic (irregular) structures where the variable parameters were the size of the surface cells in the composite structure, the thickness of the walls of the material used for 3D printing (the walls that make up the cells), and the degree of irregularity in the distribution of pores throughout the inner core of the composite. The graphical software used for automatic parametric modelling was Rhino 7, and the overlays were Grasshopper® 3D, Lunch-box 2025.4.16.0, and Kangaroo 2.5.3—see Figure 1.

Voronoy diagrams were applied based on the 3D diagram and the attractor [53]. A random point cloud was used as the initial state of the algorithm. The 3D diagram from the random point cloud emits spheres. Populate 3D should appear as the first block on the canvas, where the Region plugin expects the introduction of a perpendicular. It is best to start by preparing the perpendicular. Use the rectangle block to prepare a square of the expected side length. In the next steps, use the Bonded Surfaces block to turn the square into a full face and the Extrude, Unit Z, and Slider block set to bring it to the desired length. The square prepared in this way should be plugged into the plug area of the Populate 3D block. Other plug-ins should have sliders: Count in a slider with a value of 100 and Speed in a slider with an arbitrary value. Then, on the surface of the Voronoi 3D block, we plug the data from Populate 3D into the Points plugin and the previously prepared cube into the Box plugin. The random and quantitative arrangement of the cells can be adjusted with sliders plugged into the Populate 3D block. The next step is to create an attractor that scales the newly created cells of the 3D Voronoi diagram. The way the attractor works is related to the relationship between the distance of the cell from the ground and its size. This is done using an attractor called “Point and Circles” which is used to create a regular grid of circles which, controlled by the attractor, will additionally change their size. The work starts with the Square block, which creates a grid of squares of a specified length, width, and size across the entire structure (grid). We used the slider to set the dimensions of the grid, i.e., we plugged the slider into the Extend plugin. The second step was to create a point at each centre of the previously dimensioned squares. In this case, we used the Area block. This block is essentially used to calculate the area of a closed curve or surface, but it has an additional function, i.e., it calculates the geometric centre of the figure. The cable coming from the Square block (Cells plugin) should be plugged into the Area block. The output is a graft tree. The third stage is the construction of circles inscribed in squares using the Circle CNR block. We connect the cable coming from the Area block from the Cells connector to the Circle CNR connector. We still need to characterise the radius value of the circle Radius with a slider value of 0.5. In the fourth stage of the work, we need to create a line between a moving point at the centre of each of the circles or squares we have created. The centre is the same for both geometric figures. To do this, we use the Line block. This is a simple block that creates a line between two particular points. The first is a point from the Point hexagonal block, and the second is a point from the Area block of the Centroid plugin. The result is a line from each centre of the circle/square to a point on plane 0 (the centre of the starting square). The length of these lines is crucial to the overall principle of the Tractor algorithm, as it affects the degree of scaling of the circles. In this situation, it is first necessary to measure the distance of each individual cell to the point at plane 0. This can be done in a number of ways, but the simplest and most straightforward seems to be to place the point at the centre of the initial square using the Area block, which, in the Centroid plugin on the right, outputs a point at the centre of the square with x, y, z coordinates. The geometric centre of each cell must then be determined. The easiest way to do this is to use the Volume block, which calculates the volume. It also calculates the spatial centroid of the enclosed geometry. The next block needed is Distance, a simple block that calculates the distance in a straight line between two points. The data from the Centroid plugin of the Volume block should be plugged into the Point A plugin, and the data from the Centroid plugin, but not from the Area block, should also be plugged into the Point B plugin. This will provide the distance of all cells from the ground. These distances are used to characterise the attractor. The structure of the attractor is classical. On the Remap Numbers page, enter the data for the corresponding blocks one by one. The Volume plugin should be filled with distances from the Distance block, while the Secure plugin should be filled with data from the Bonus block, which should be placed on the canvas beforehand and distances plugged into it. The last plugin, Target, should be defined as a number domain. For this, you will need the Dominant Construct block and the two Dominant End sliders. Area should be set to 1, while Dominant Start should be set to 0.1 or slightly higher. This will prevent the cells from being overscaled. The final step is to scale the cells according to the attractor principle. Place the Scale block on the canvas and plug the data from the Voronoi 3D block into the Geometry plugin. Plug the data from the Volume block into the Centre plugin and the data from the Remap Numbers plugin into the Factor plugin.

Finally, a multifunctional, gradient, and heterogeneous cellular structure was obtained.

In the study, a 50 × 50 × 50 mm^3^ uniform and graded irregular monolayer structure and a 50 × 50 × 100 mm^3^ uniform and graded irregular bilayer structure were designed by controlling the seed distribution and wall thickness of the cellular composite core.

**Figure 2:**
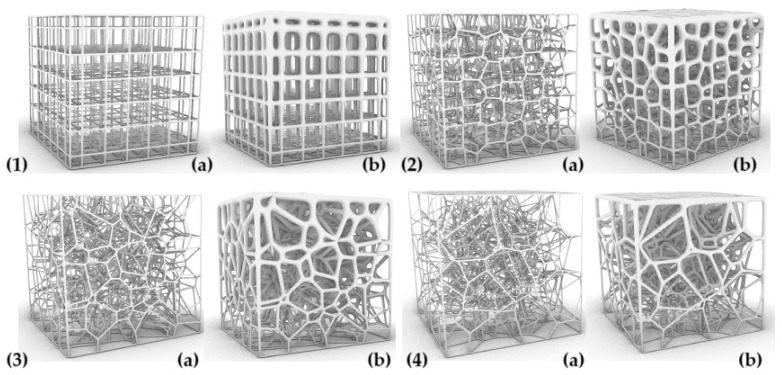
Influence of changes in R and D values on the shape of the inner core structure in the form of a Voronoi cell with dimension S = 8 mm: (**1a**) R = 0 and D = 0.2 mm, (**1b**) R = 0 and D = 0.2 mm and D = 0.8 mm, (**2a**) R = 1 and D = 0.2 mm, (**2b**) R = 1 and D = 0.2 mm and D = 0.8 mm, (**3a**) R = 2 and D = 0.2 mm, (**3b**) R = 2 and D = 0.2 mm and D = 0.8 mm, (**4a**) R = 4 and D = 0.2 mm, (**4b**) R = 4 and D = 0.2 mm and D = 0.8 mm.

**Figure 3:**
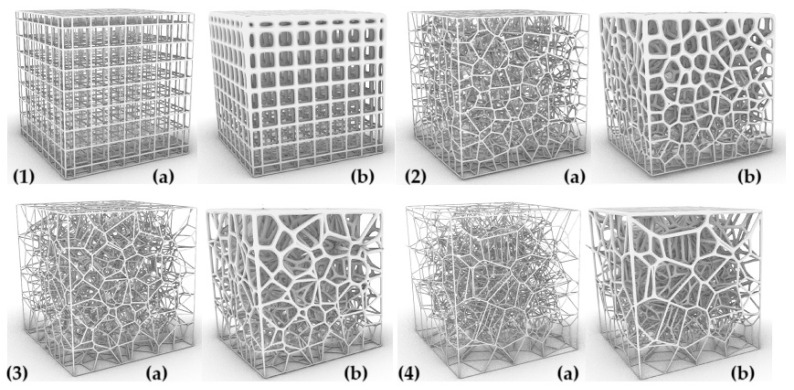
The influence of changes in R and D values on the shape of the inner core structure in the form of a Voronoi cell with a single cell dimension of S = 6 mm: (**1a**) R = 0 and D = 0.2 mm, (**1b**) R = 0 and D = 0.2 mm and D = 0.8 mm, (**2a**) R = 1 and D = 0.2 mm, (**2b**) R = 1 and D = 0.2 mm and D = 0.8 mm, (**3a**) R = 2 and D = 0.2 mm, (**3b**) R = 2 and D = 0.2 mm and D = 0.8 mm, (**4a**) R = 4 and D = 0.2 mm, (**4b**) R = 4 and D = 0.2 mm and D = 0.8 mm.

**Figure 4:**
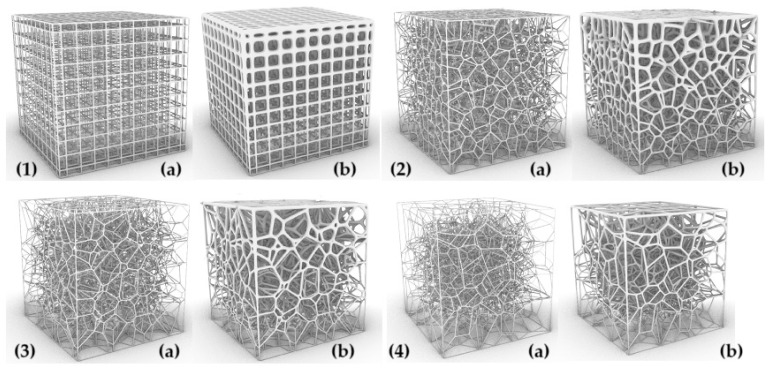
The influence of changes in R and D values on the shape of the inner core structure in the form of a Voronoi cell with a single cell dimension of S = 5 mm: (**1a**) R = 0 and D = 0.2 mm, (**1b**) R = 0 and D = 0.2 mm and D = 0.8 mm, (**2a**) R = 1 and D = 0.2 mm, (**2b**) R = 1 and D = 0.2 mm and D = 0.8 mm, (**3a**) R = 2 and D = 0.2 mm, (**3b**) R = 2 and D = 0.2 mm and D = 0.8 mm, (**4a**) R = 4 and D = 0.2 mm, (**4b**) R = 4 and D = 0.2 mm and D = 0.8 mm.

**Figure 5:**
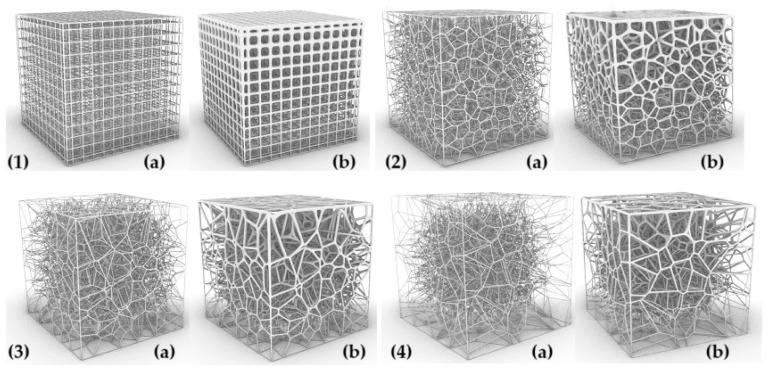
The influence of changes in R and D values on the shape of the inner core structure in the form of a Voronoi cell with a single cell dimension of S = 4mm: (**1a**) R = 0 and D = 0.2 mm, (**1b**) R = 0 and D = 0.2 mm and D = 0.8 mm, (**2a**) R = 1 and D = 0.2 mm, (**2b**) R = 1 and D = 0.2 mm and D = 0.8 mm, (**3a**) R = 2 and D = 0.2 mm, (**3b**) R = 2 and D = 0.2 mm and D = 0.8 mm, (**4a**) R = 4 and D = 0.2 mm, (**4b**) R = 4 and D = 0.2 mm and D = 0.8 mm.

**Figure 6:**
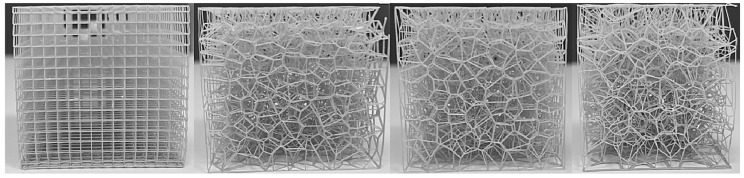
Example printouts (inner core of the composites) of the samples: regular—the first one on the left, and irregular—the next three examples for D = 0.2 mm.

**Figure 7:**
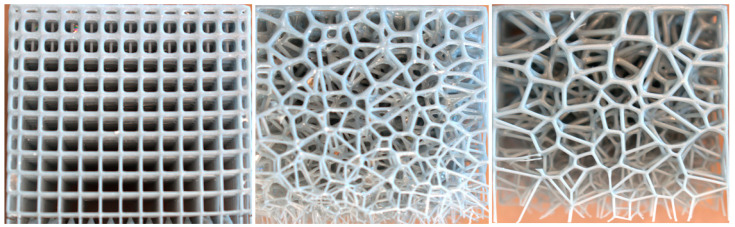
Sample prints (inner core of composites) of samples: regular—first on the left and irregular—three consecutive examples for wall thicknesses (D) from 0.2 mm to 0.8 mm.

The parameters characterizing the variability of the geometry of the designed cellular composites are in the first step—the regularity of the pore distribution (R) for R = 0 (regular) and for R = 1, R = 2, R = 4 (irregular) combined with the variable size of the air cells in the composite structure—for 8 mm, 6 mm, 5 mm, and 4 mm and the variable irregularity of the wall thickness of the struts in the composite (D) for the wall thickness D = 0. 2 for one test and D ranging from 0.2 to 0.8 for the second test. A total of 32 types of cellular composite tests were performed in three replicates each (Figure 2, Figure 3, Figure 4, Figure 5, Figure 6 and Figure 7).

**Figure 8:**
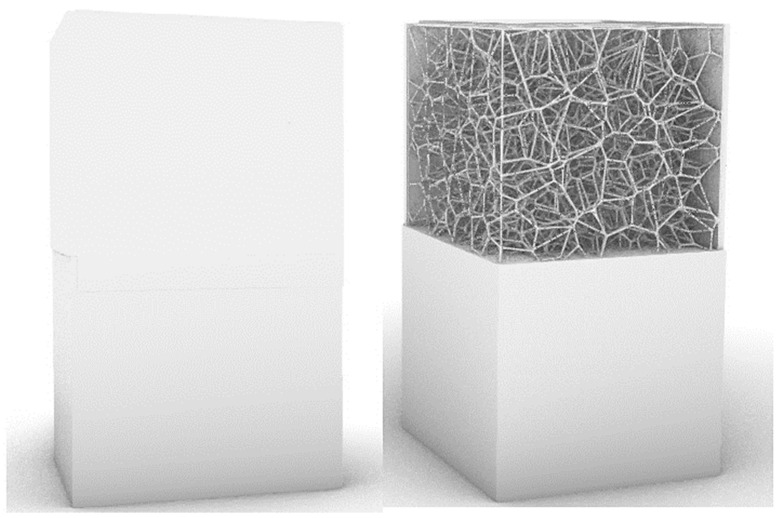
Examples of two-layer cellular composites.

In a second step, the layering parameter (W) was used to determine the layering of the produced cellular composites. Single- and two-layer composites (Figure 8) were designed and printed for the previously assumed parameters. The tests were conducted for the samples with the lowest thermal conductivity coefficients, i.e., for the air cell size (P) of 4 mm and with the regularity of the pore distribution (R) for R = 0–4 (regular and irregular) and variable irregularity of the wall thickness of the struts in the composite (D) for the wall thickness D = 0.2 mm for one sample and D in the range from 0.2 mm to 0.8 mm for the second sample.

### 2.2. Experimental Section

The experiment was performed according to ISO 9869-1:2014 [52] using a test rig available at the Faculty of Mechanical and Energy Engineering, Wroclaw University of Technology, in the Department of Energy Conversion Engineering [54,55]. The set-up consisted of an Aisberg LP15 C15 freezer (MELIS, Poznań, Poland), a heat-flow sensor FHF04SC (Hukseflux Thermal Sensors B.V., Delft, The Netherlands), 4 thermocouples (K-type thermocouples), and a temperature and heat-flow recorder. The values of the thermal-resistance coefficient (R) and the heat-transfer coefficient (λ) were calculated according to the “average method” proposed in ISO 9869 [52].

The exact method of performing the test and the schematic diagram and photographs of the test rig are described in the author’s previous publications [56,57,58,59,60].

## 3. Results and Discussion

The aim of the study was to evaluate the thermal properties of the printed composites for the potential use of the materials as thermal insulation, according to ISO 9869-1:2014 [52]. The results obtained were compared according to the size and degree of irregularity of the air cells in the composite and the number of layers and thickness of the composite. In each case, three series of measurements were taken and averaged under stabilised temperature conditions. The average temperature difference between the outside and inside of the cooling chamber was 10 °C.

Statistical analyses were performed using STATISTICA 13 (TIBCO Statistica, Palo Alto, CA, USA). A significance level of *p* ≤ 0.05 was used, in line with common practice in thermal insulation studies. Measures of position and dispersion were first determined, and their pooled results are presented in Table 1, Table 2, Table 3 and Table 4. Analysis of the significance level (*p*) values in Table 5 and Table 6 shows that values less than 0.05 indicate a significant effect of the experimental input variables on the value of the thermal properties obtained in the experiment of the tested materials produced by 3D mSLA printing technology.

As shown in Table 1, the thermal conductivity coefficient (λ) values of the 50 mm-thick single-layer composite samples ranged from 0.034 to 0.066 W/(m·K). The mean value was 0.045 W/(m·K), and the standard deviation was 0.0073 W/(m·K). The value of the thermal conductivity coefficient for half of the tested samples did not exceed 0.044 W/(m·K). The tested samples had a high kurtosis value of K = 0.673, which means that the results were clustered around the mean. The thermal-resistance coefficient (R) values of the 50 mm-thick monolayer composite samples (Table 2) ranged from 0.757 to 1.471 (m^2^·K) W. The mean value was 1.127 (m^2^·K)/W, and the standard deviation was 0.167 (m^2^·K)/ W. The value of the thermal resistance coefficient for half of the samples tested did not exceed 1.125 (m^2^·K)/W.

For single- and two-layer samples with a composite thickness of 100 mm, the thermal conductivity coefficient (λ) values (Table 3) ranged from 0.026 to 0.049 W/(m·K), with a mean of 0.036 W/(m·K) and a standard deviation of 0.007 W/(m·K). In this case, the value of the thermal conductivity coefficient for half of the samples tested did not exceed 0.035 W/(m·K). The values of the thermal-resistance coefficient (R) of the single- and two-layer composite samples with a thickness of 100 mm (Table 4) ranged from 2.04 to 3.92 (m^2^·K) W. The mean value was 2.88 (m^2^·K)/W, and the standard deviation was 0.50 (m^2^·K). The value of the thermal-resistance coefficient for half of the tested samples did not exceed 2.86 (m^2^·K)/W.

The results of the analysis of variance (Table 5 and Table 6) showed that there was an effect of air cell size, air cell orientation irregularity, and variation in the thickness of the internal struts in the composite and the thickness of the composite and the number of layers in the composite on the thermal properties of the 3D printed insulation barriers, as evidenced by the *p*-value. The statistical significance of the interaction of the linear factors was also demonstrated by the high value of the power of influence (F). Cell size and the degree of irregularity and layering in the cellular composites were shown to be highly dominant factors compared to the other input factors.

The results of the analysis of variance (Table 5 and Table 6) showed that the size of the air cells, the inhomogeneity of their orientation and the variation of the thickness of the internal reinforcements in the composite, as well as the thickness of the composite and the number of its layers, affected the thermal properties of the insulation barriers produced by 3D printing technology, as confirmed by the *p*-value. The statistical significance of the linear factors was also confirmed by the high value of the effect size (F). The cell size and the degree of inhomogeneity and layering in the cellular composites proved to be the most dominant factors compared to other input variables. The study also confirmed the statistical significance of the interaction of linear factors. It was shown that the degree of inhomogeneity of the air cell orientation used in the internal structure of the composite is a factor that clearly dominates over the other input variables. The interactions of the input variables play a marginal role compared to the linear factors, which means that for each input variable, there is an optimal type of composite structure, independent of the other factors. In summary, in order to obtain optimal insulation properties of the produced composites, the most favorable values of air cell size (S), the optimal level of air cell distribution inhomogeneity (R), and wall thickness (D) in the internal structure, as well as the best value of the number of layers (W) in the composite, can be determined. Each input variable is set independently.

Preliminary analysis of the data, as well as their nature and origin, allows us to conclude that the appropriate model of the relationship in each of the four cases considered is the response surface; that is, the fitting of an appropriate quadratic polynomial (of degree 2). Such a polynomial would, for example, if its input quantities were certain A and B, be a certain linear combination of a constant, the linear elements A and B, and the quadratic elements A^2^, AB, and B^2^. Non-significant elements (with a *p*-value > 0.05) can generally be excluded from the model, as this usually results in only a small loss of model accuracy.

**Figure 9:**
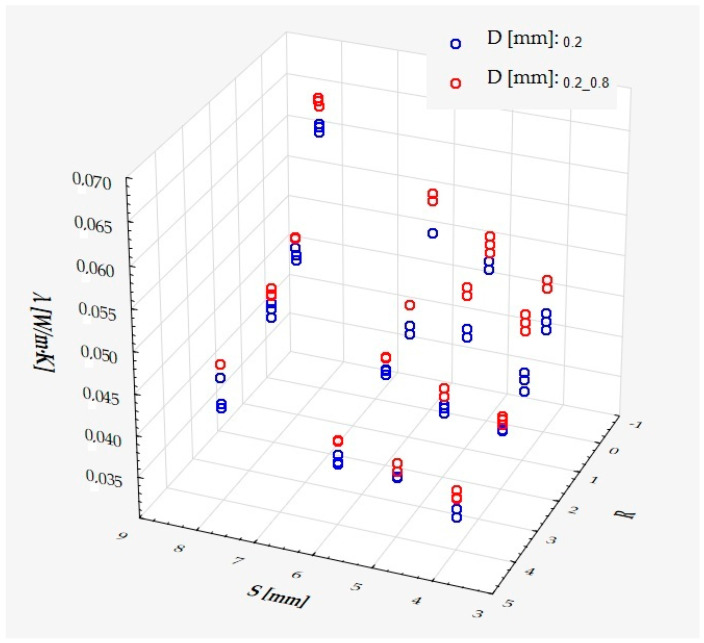
Thermal-conductivity coefficient as a function of S, D, and R.

For the obtained values of the thermal conductivity (λ) for the samples with a composite thickness of 50 mm, the following surface model has been fitted:

Y = 0.030708 + 0.003394·S + 0.010117·D − 0.006006·R + 0.001165·R^2^ − 0.000393·SR + ε (1)

which describes R^2^ = 94.4% of the variance of the dependent variable. In the above formula, all coefficients are significantly different from zero (*p* < 0.00001 in each case), and ε denotes a random component with a normal distribution, a mean equal to zero, and a standard deviation equal to 0.00177—this quantity is the standard error of estimation. The formula shows that the dependent variable increases on average with S and D, while it decreases with an increase in R, at least in the range of values considered. These relationships can also be seen in the following three-dimensional scatterplot—Figure 9.

**Figure 10:**
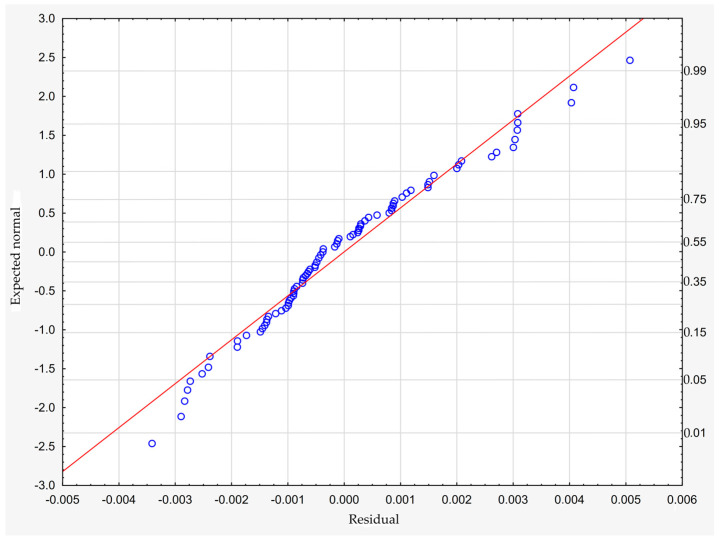
Normality plot of the residuals from the model.

**Figure 11:**
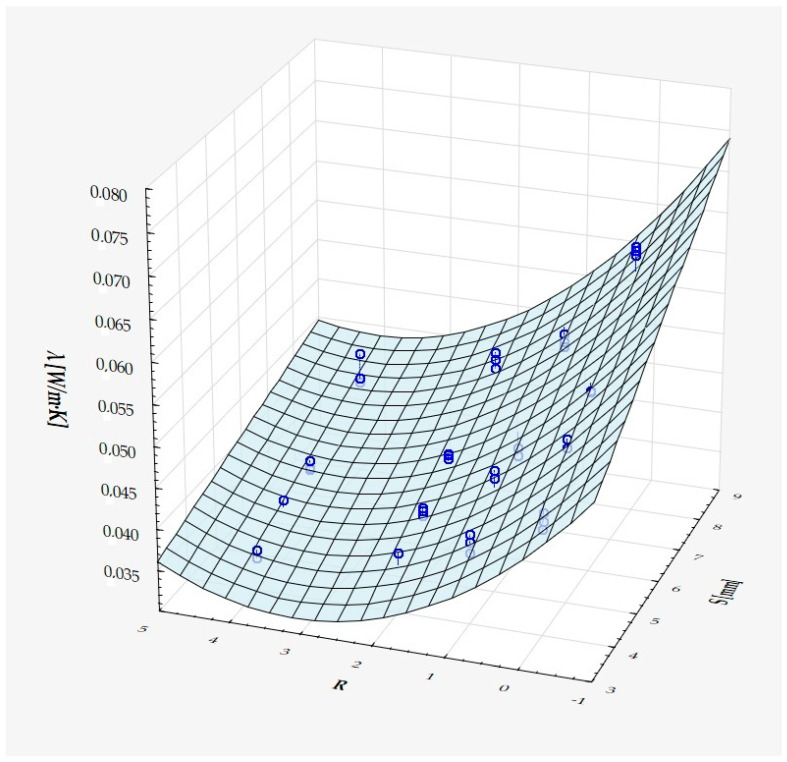
Matching surface for D = 0.2 mm.

**Figure 12:**
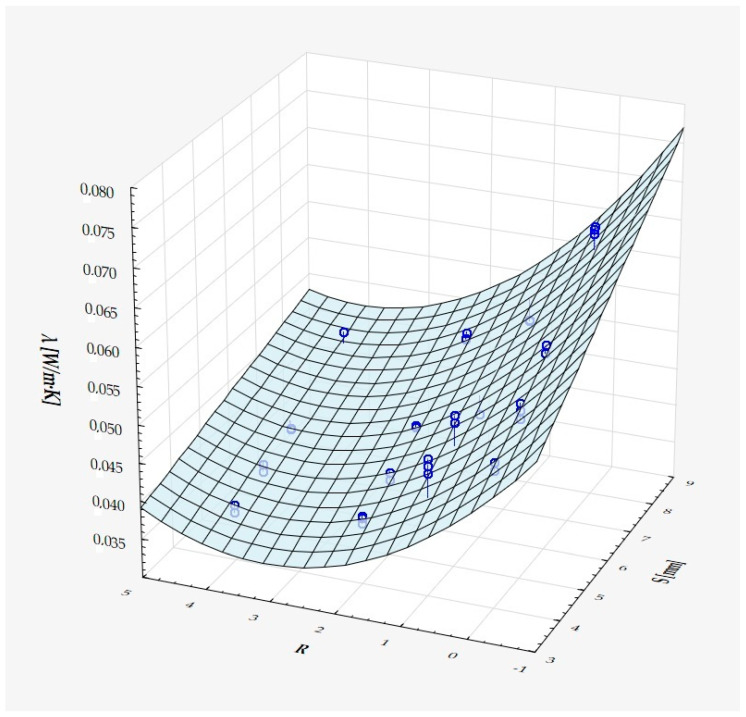
Matching surface for D = 0.2–0.8 mm.

The model can be considered very good due to the high value of the coefficient of determination R^2^ and the small number of coefficients (6), and the assumption of normality of the distribution of the model residuals is only slightly violated (*p* = 0.024 in the Shapiro–Wilk normality test)—Figure 10, Figure 11 and Figure 12.

**Figure 13:**
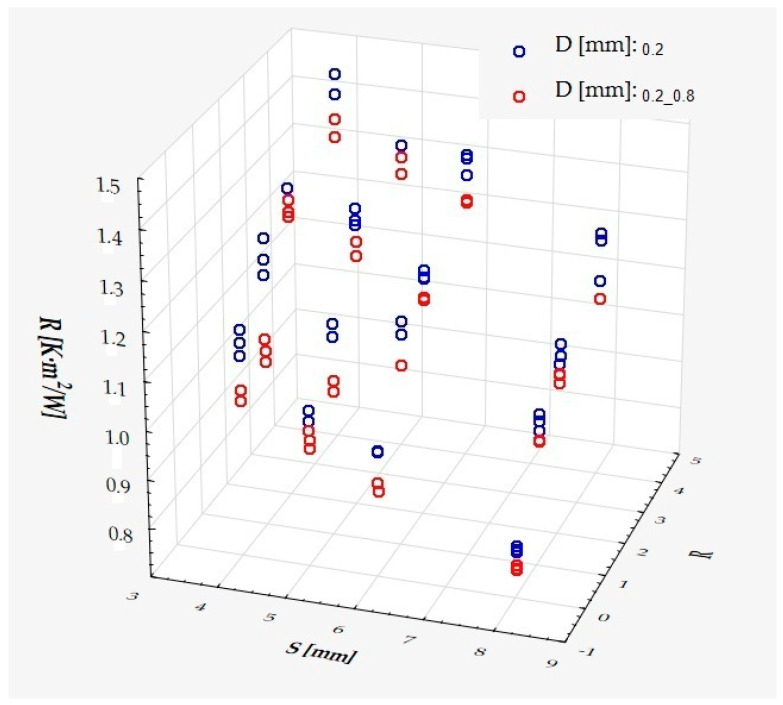
The coefficient of thermal resistance as a function of S, D, and R.

For the obtained values of the thermal-resistance coefficient (R) for the samples with a composite thickness of 50 mm, the following surface model was fitted:

Y = 1.388446 − 0.062675·S − 0.249115·D + 0.168943·R − 0.020889·R2 + ε (2)

which describes R^2^ = 95.4% of the variance of the dependent variable. In the above formula, all coefficients are significantly different from zero (*p* < 0.00001 in each case), and ε denotes a random component with a normal distribution, a mean of zero, and a standard deviation of 0.03643—this quantity is the standard error of the estimate. It can be seen from the formula that, on average, the dependent variable decreases as S and D increase, while it increases as R increases, at least in the range of values considered. These relationships can also be seen in the following three-dimensional scatterplot—Figure 13.

**Figure 14:**
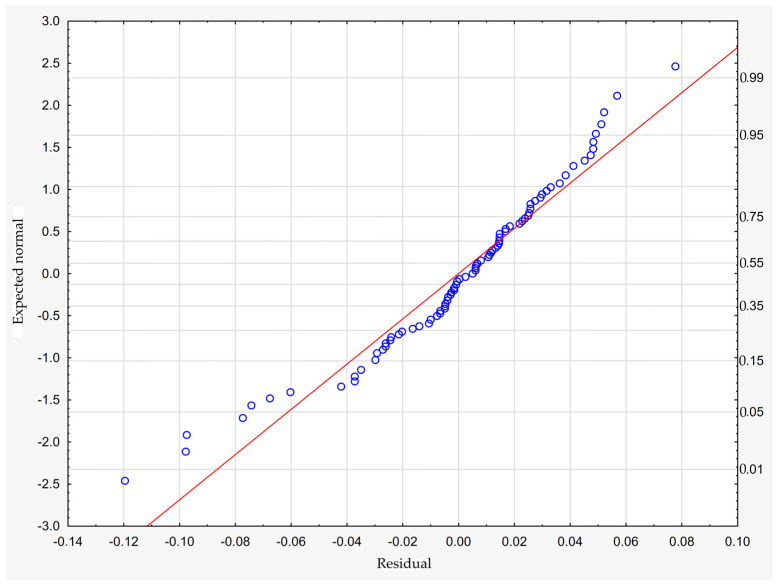
Normality plot of the residuals from the model.

**Figure 15:**
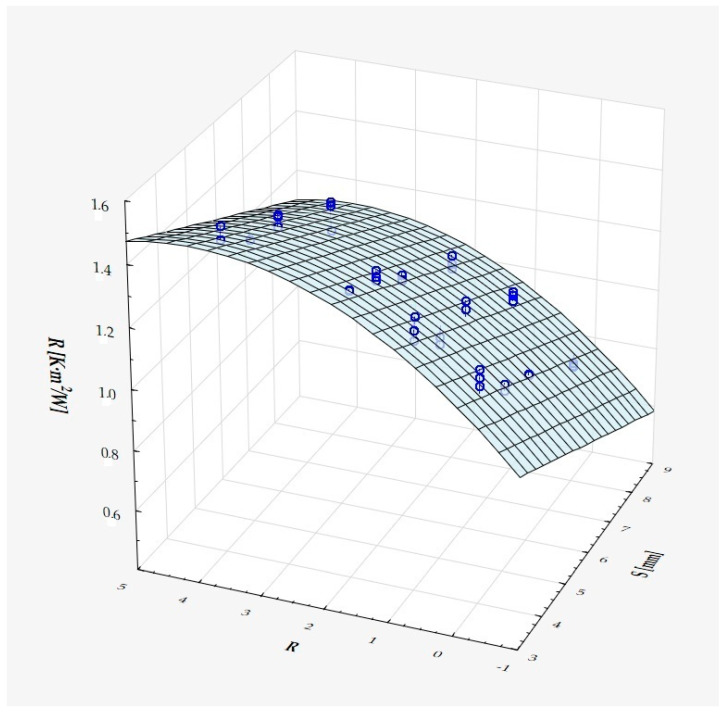
Matching surface for D = 0.2 mm.

**Figure 16:**
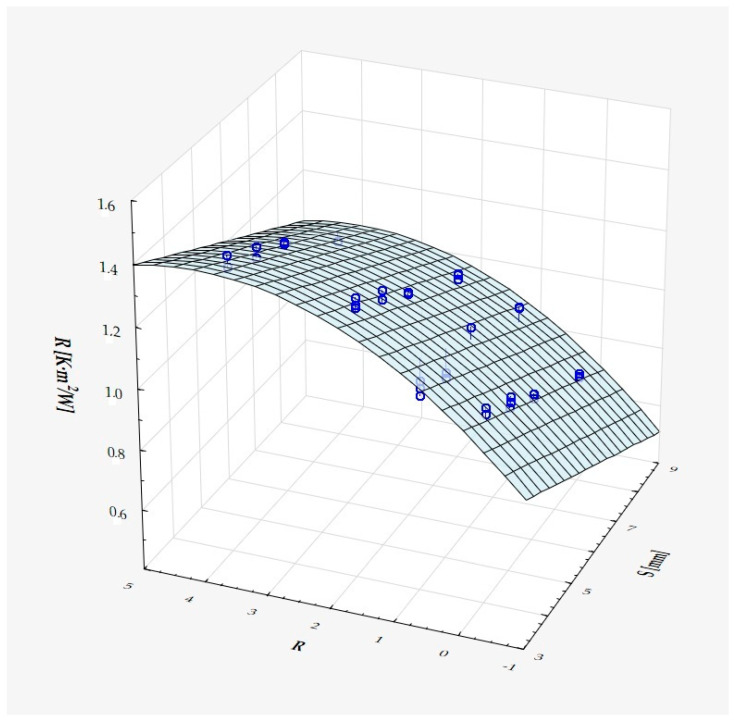
Matching surface for D = 0.2–0.8 mm.

The model can be considered very good due to the high value of the coefficient of determination R^2^ and the small number of coefficients (5), despite the fact that the assumption of normality of the distribution of the model residuals is slightly violated (*p* = 0.0008 in the Shapiro–Wilk normality test)—Figure 14, Figure 15 and Figure 16.

**Figure 17:**
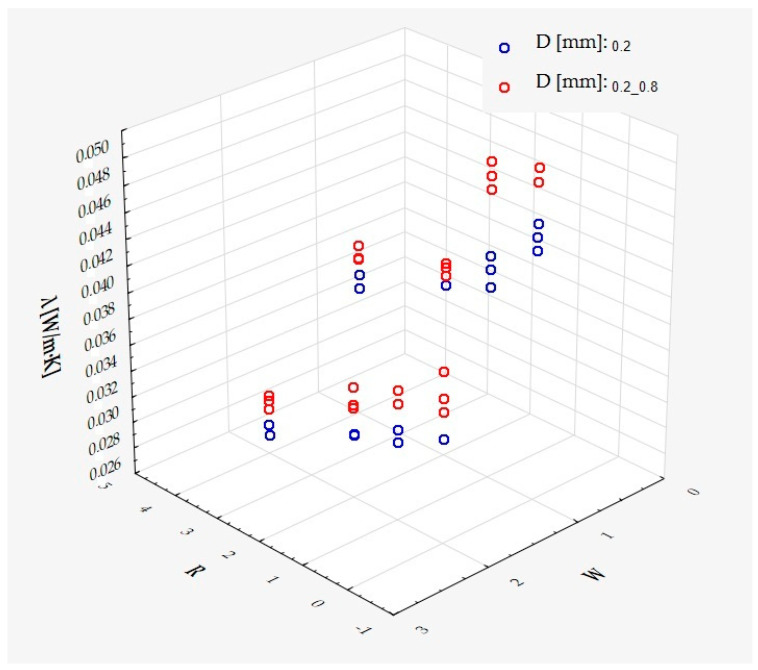
Thermal-conductivity coefficient as a function of D, R, and W.

For the obtained values of the thermal-conductivity coefficient (λ) for the 100 mm-thick single-layer and double-layer composite samples, the ordinary linear regression model was fitted. All coefficients of the second-degree members were found to be insignificant:

Y = 1.670290 − 0.833797·D + 0.172714·R + 0.801948·W + ε(3)

where R^2^ = 97.4% of the variance of the dependent variable. In the above formula, all coefficients are significantly different from zero (*p* < 0.00001 in each case), and ε denotes a random component with a normal distribution, a mean of zero, and a standard deviation equal to 0.084—this quantity is the standard error of estimation. From the formula, we can see that the dependent variable increases on average with R and W and decreases with an increase in D, which can also be seen in the following three-dimensional scatterplot—Figure 17.

**Figure 18:**
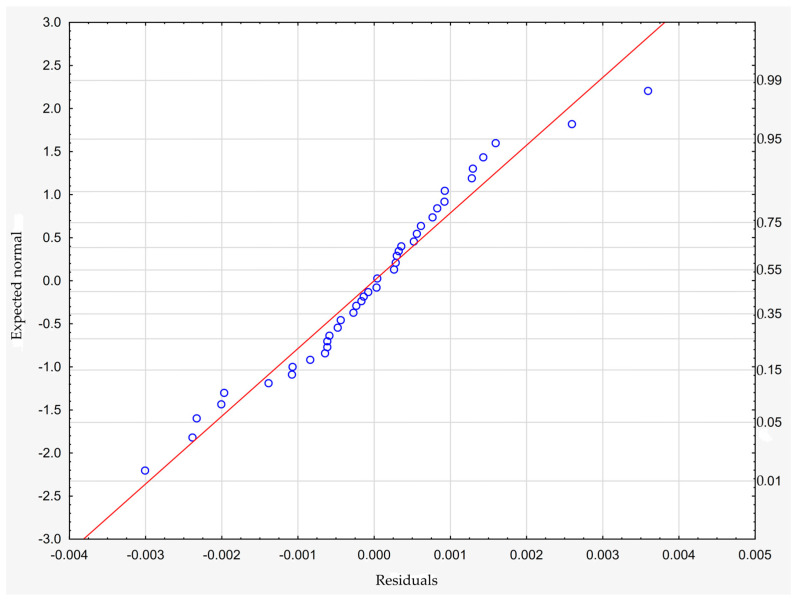
Normality plot of the residuals from the model.

**Figure 19:**
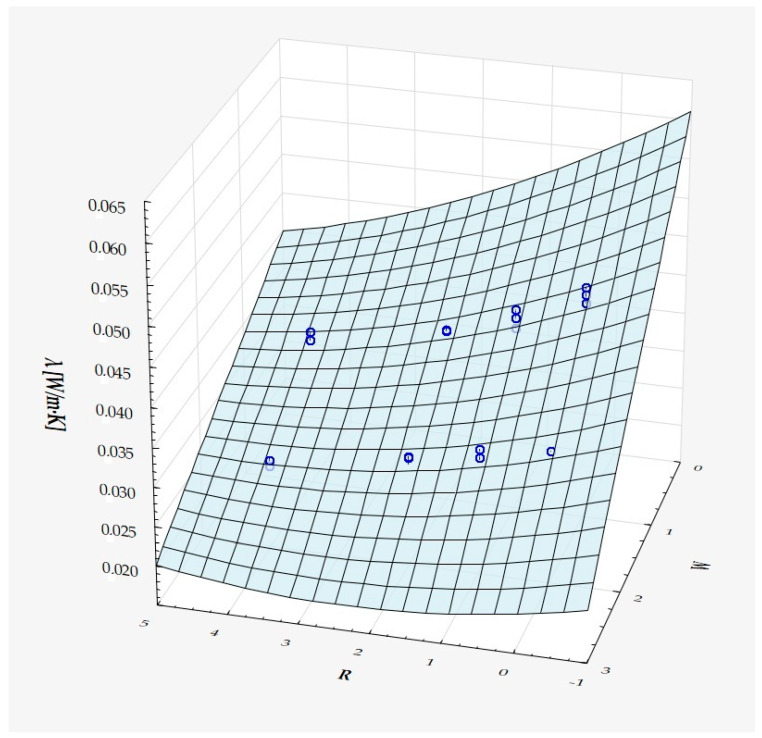
Matching surface for D = 0.2 mm.

**Figure 20:**
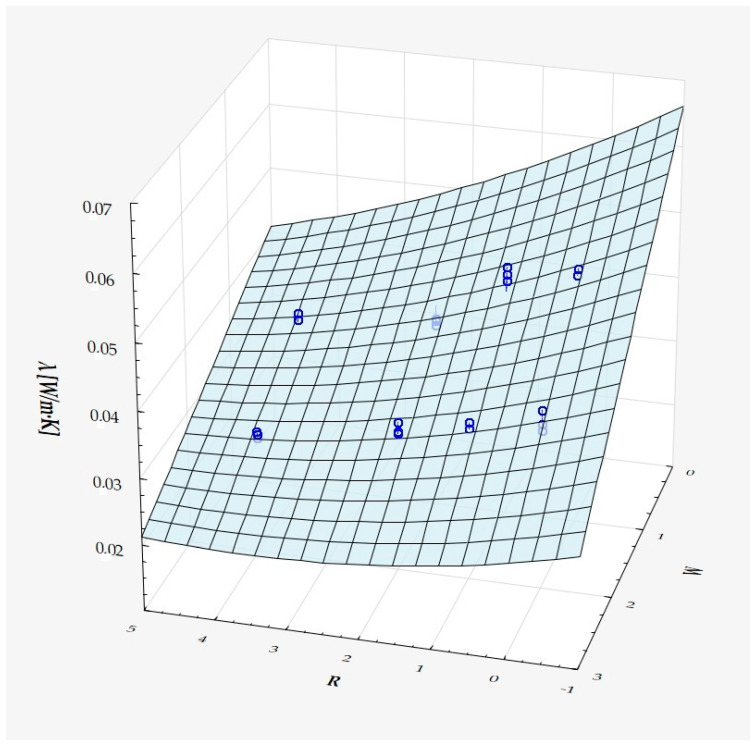
Matching surface for D = 0.2–0.8 mm.

The model can be considered very good due to the high value of the coefficient of determination R^2^ and the small number of coefficients (4), and the assumption of normality of the distribution of the model residuals is only slightly violated (*p* = 0.031 in the Shapiro–Wilk normality test)—Figure 18, Figure 19 and Figure 20.

**Figure 21:**
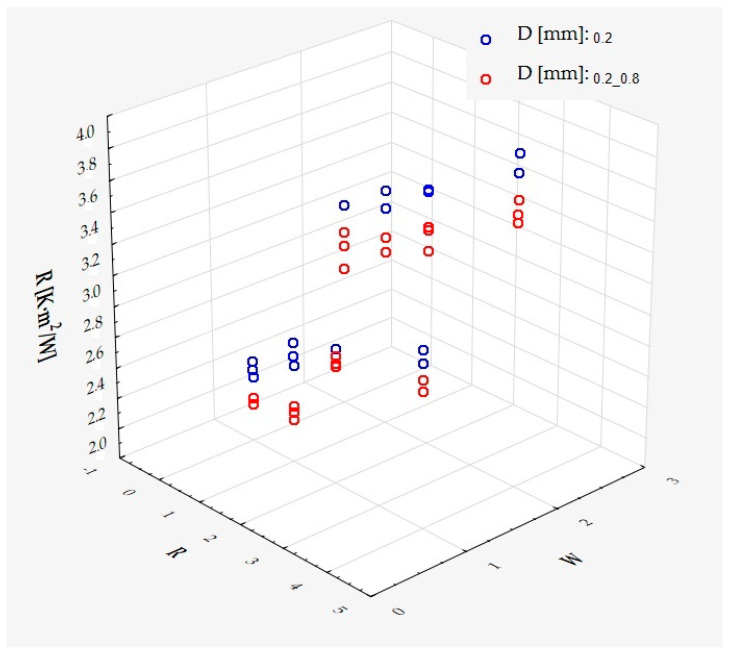
The coefficient of thermal resistance as a function of D, R, and W.

An ordinary linear regression model was fitted to the obtained values of the thermal-resistance coefficient (R) for 100 mm-thick composite monolayer and bilayer specimens, as all coefficients of second-order members were found to be insignificant:

Y = 1.670290 − 0.833797·D + 0.172714·R + 0.801948·W + ε (4)

where R^2^ = 97.4% of the variance of the dependent variable. In the above formula, all coefficients are significantly different from zero (*p* < 0.00001 in each case), and ε denotes a random component with a normal distribution, a mean of zero, and a standard deviation equal to 0.084—this quantity is the standard error of estimation. From the formula, we can see that the dependent variable increases on average with R and W and decreases with an increase in D, which can also be seen in the following three-dimensional scatterplot—Figure 21.

**Figure 22:**
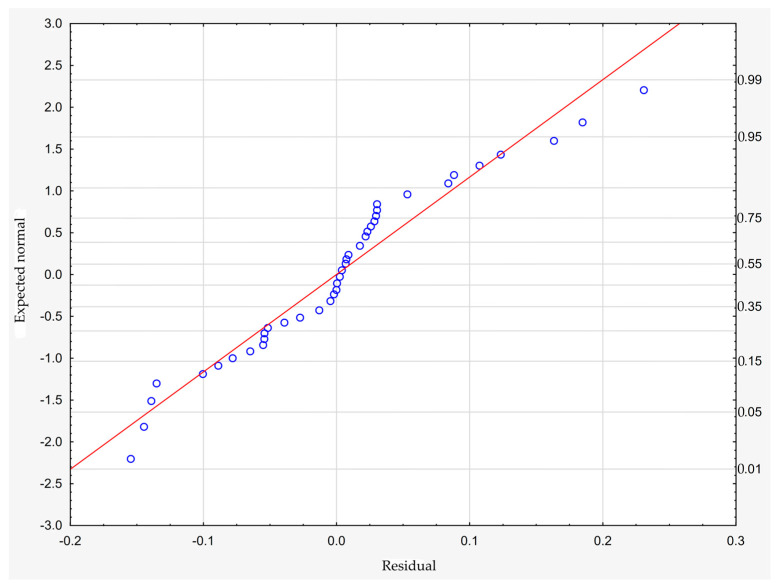
Normality plot of the residuals from the model.

**Figure 23:**
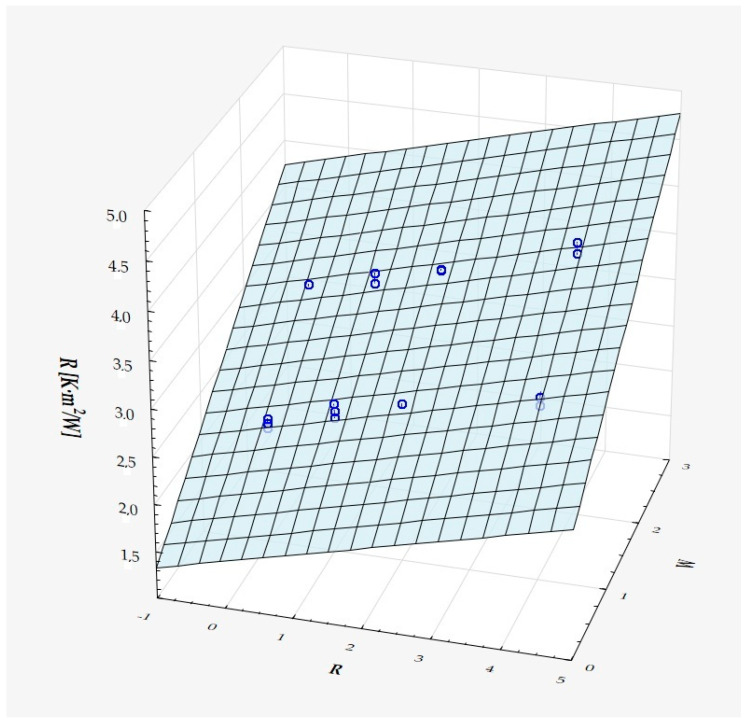
Matching surface for D = 0.2 mm.

**Figure 24:**
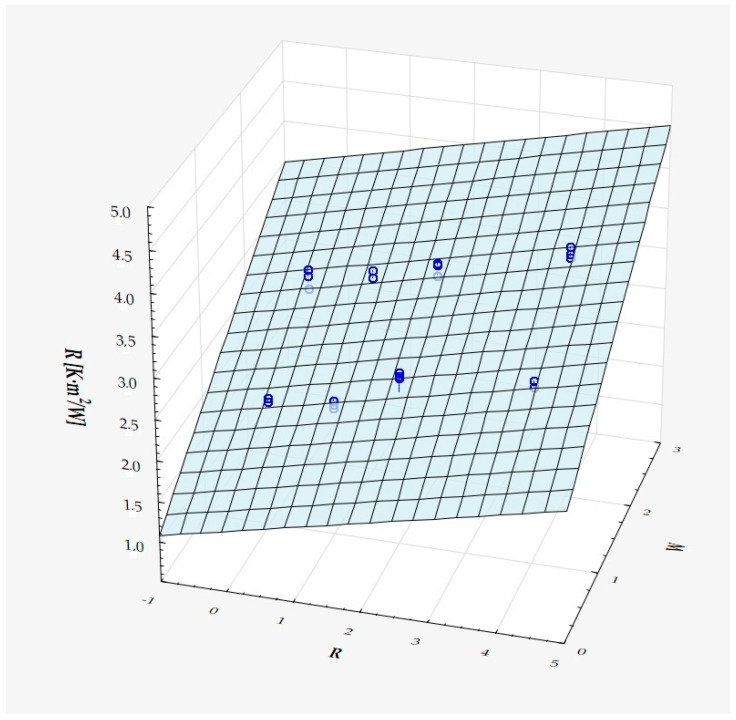
Matching surface for D = 0.2–0.8 mm.

The model can be considered very good due to the high value of the coefficient of determination R^2^ and the small number of coefficients (4), and the assumption of normality of the distribution of the model residuals is only slightly violated (*p* = 0.031 in the Shapiro–Wilk test of normality)—Figure 22, Figure 23 and Figure 24.

It has been shown that the size of the air cells influences the thermal conductivity coefficient and the thermal resistance. Figure 9, Figure 11, Figure 12, Figure 13, Figure 15 and Figure 16 show the obtained difference between the parameters of the air cell size in the Voronoi cell structure for samples with a thickness of 50 mm. Samples with cell size S = 4 mm had the lowest thermal-conductivity coefficient (λ = 0.034 W/(m·K) and, thus, the highest thermal-resistance coefficient (R = 1.471(m^2^·K)/W) than samples with air cell size S = 5, 6, 8 mm. The difference in the values obtained was large in this case.

The results of the analysis of variance also showed that the wall thickness of the Voroni structure has an influence on the obtained values of the thermal conductivity coefficient (λ) and thermal resistance. Figure 9, Figure 11, Figure 12, Figure 13, Figure 15, Figure 16, Figure 17, Figure 19, Figure 20, Figure 21, Figure 23 and Figure 24 show the relationship between the wall thicknesses of the composite structure assumed in the experiment and the data obtained. Samples with a wall thickness of D = 0.2 mm had a lower coefficient of thermal conductivity (λ) and, thus, a higher thermal resistance than samples with D = 0.2 mm and D = 0.8 mm, and the difference in the results obtained was moderately large.

The results of the analysis of variance also showed that the degree of irregularity of the air cell arrangement in the internal structure of the composite has an influence on the obtained values of the thermal conductivity coefficient (λ) and thermal resistance. Figure 9, Figure 11, Figure 12, Figure 13, Figure 15, Figure 16, Figure 17, Figure 19, Figure 20, Figure 21, Figure 23 and Figure 24 show the relationship between the degree of structure irregularity assumed in the experiment and the data obtained. Samples with a degree of cell arrangement irregularity of R = 4 had the lowest thermal conductivity coefficient (λ) and, thus, the highest thermal resistance compared to samples with a degree of irregularity of R = 0 and R = 1 and 2, and the difference in the results obtained was moderately large.

The analysis also showed the influence of the number of layers (W) in the composite on the values obtained for the coefficients of thermal conductivity and thermal resistance. Figure 17, Figure 19, Figure 20, Figure 21, Figure 23 and Figure 24 show a graphical comparison of the results obtained for different numbers of layers used in the experiment. Samples with a number of layers W = 2 had a lower coefficient of thermal conductivity and, therefore, a higher thermal resistance than samples with a number of layers W = 1. The difference in the results obtained was moderately large.

## 4. Conclusions

Design inspired by nature leads to the creation of high-strength structures with minimal material consumption. Examples include structures that resemble organic tissues, shells, and crystal lattices. Popular examples include the structure of mussel shells, spider webs, and porous bone structures, which inspire modern composite materials with high strength and elasticity. Voronoi tessellations and other biomimetic patterns can be used to control key physical properties of materials. Methods for modifying these structures include parameterization of algorithm input parameters, topological analysis, and geometric optimization. Biomimetics is widely used in aerospace, biomaterials, and architecture. Voronoi tessellations are a promising tool for the design of advanced materials and structures. The ability to control their properties opens up new perspectives in engineering and science, and further development of the technology can lead to new, innovative applications. Integration with computational optimization methods and artificial intelligence will allow even better adaptation of designed structures to specific functional requirements.

Despite the great potential, the control of the properties of Voronoi tessellation and biomimetic patterns faces challenges related to the precise control of the geometry and its macroscale effects. Future research should focus on the use of artificial intelligence and optimization algorithms to automate the process of generating structures with desired properties. In addition, the development of 3D printing allows experimental testing of the generated patterns, which allows their adaptation to real conditions of use.

The lowest thermal conductivity coefficients were obtained for prototype insulating partitions with a 100 mm-thick Voroni cell structure with: wall thickness D = 0.2 mm, cell size S = 4 mm, number of structural layers n = 2, and degree of irregularity R = 4. The lowest possible thermal conductivity of the insulation was 0.026 W/(m·K), and the highest possible thermal resistance was 3.92 (m^2^·K)/W. According to the PN-EN ISO 9229:2020-12 standard [54], the thermal conductivity coefficient of thermal insulation materials should not exceed 0.065 W/(m·K). Analysis of the results showed that, for most of the samples, the required value of the thermal conductivity coefficient was achieved. The prototype of an insulating partition with a Voronoi structure is characterized by good insulation parameters. Statistical analysis of the experimental results allowed the composition of the composite to be optimized according to the criteria adopted.

The results obtained led the author to plan further research on the cellular composites analyzed in the article, such as the determination of their mechanical properties (including compressive and impact strength). The author’s future research directions also include optimizing the thermal and mechanical properties of insulation materials produced using 3D technology, as well as using renewable or biodegradable raw materials as additional fibres added to the composite matrix. In order to lead innovation in multifunctional and intelligent insulation materials, the improvement of printing processes in this field currently seems to be crucial.

## Figures and Tables

**Table 1:** Descriptive statistics for the thermal conductivity coefficient (λ) for the 50 mm-thick single-layer composite samples (M—mean, Me—mean, Min—Min; Max—Max; SD—Std Deviation; Sk—Skewness; K—Kurtosis).

	M	Me	Min	Max	SD	Sk	K
λ, W/(m·K)	0.045	0.044	0.034	0.066	0.0073	0.901	0.673

**Table 2:** Descriptive statistics for the coefficients of thermal resistance (R) of 50 mm-thick composite samples (M—mean; Me—mean; Min—minimum; Max—maximum; SD—standard deviation; Sk—skewness; K—kurtosis).

	M	Me	Min	Max	SD	Sk	K
R, (m^2^·K)/W	1.127	1.125	0.757	1.471	0.167	−0.178	−0.575

**Table 3:** Descriptive statistics for the thermal conductivity coefficient (λ) for the 100 mm-thick the for single-, two -layer composite samples (M—mean, Me—mean, Min—Min; Max—Max; SD—Std Deviation; Sk—Skewness; K—Kurtosis).

	M	Me	Min	Max	SD	Sk	K
λ, W/(m·K)	0.036	0.035	0.026	0.049	0.007	0.542	−0.529

**Table 4:** Descriptive statistics for the coefficients of thermal resistance (R) of 50 mm-thick composite samples for single and double layers (M—mean; Me—mean; Min—minimum; Max—maximum; SD—standard deviation; Sk—skewness; K—kurtosis).

	M	Me	Min	Max	SD	Sk	K
R, (m^2^·K)/W	2.88	2.86	2.04	3.92	0.50	0.10	−0.72

**Table 5:** Quantitative assessment of the main effects and the effects of interactions—identification of the impact of dominant and statistically significant input factors on the dependent variable λ and R for single-layer composite specimens 50 mm-thick (SS—Sum of Squares, df—Degrees of Freedom, MS—Mean Square, F—F Coefficient, *p*—Significance Level).

Symbol That Identifies the Input Factors and Their Interactions	SS	df	MS	F	*p*
λ, W/(m·K)					
Absolute term	1	0.1979	0.197997	422,809.7	0.000
S	3	0.0016	0.000520	1111.3	0.000
D	1	0.000221	0.000221	472.1	0.000
R	3	0.002895	0.000965	2060.7	0.000
S*D	3	0.000004	0.000001	3.1	0.034
S*R	9	0.00024	0.000027	57.6	0.000
D*R	3	0.000023	0.000008	16.5	0.000
S*D*R	9	0.00003	0.000003	6.8	0.000
Error	64	0.00003	0.000		
General	95	0.005			
R, (m^2^·K)/W					
Absolute term	1	121.9009	121.90	371,490.6	0.000
S	3	0.8331	0.278	846.2	0.000
D	1	0.1340	0.134	408.5	0.000
R	3	1.5610	0.52	1585.7	0.000
S*D	3	0.0084	0.003	8.5	0.000
S*R	9	0.0533	0.006	18.0	0.000
D*R	3	0.0086	0.003	8.7	0.000
S*D*R	9	0.0179	0.002	6.1	0.000
Error	64	0.0210	0.0003		
General	95	2.6373			

**Table 6:** Quantitative assessment of the main effects and the effects of interactions—identification of the impact of dominant and statistically significant input factors on the dependent variable λR and U for single-layer and two-layer composite specimens with a thickness of 100 mm (SS—Sum of Squares, df—Degrees of Freedom, MS—Mean Square, F—F Coefficient, *p*—Significance Level).

Symbol That Identifies the Input Factors and Their Interactions	SS	df	MS	F	*p*
λ, W/(m·K)					
Absolute term	1	0.061408	0.061408	110,436.1	0.000
D	1	0.000128	0.000128	229.7	0.000
R	3	0.000520	0.000173	311.5	0.000
W	1	0.001227	0.001227	2206.2	0.000
D*R	3	0.000021	0.000007	12.7	0.000
D*W	1	0.000003	0.000003	5.5	0.025
R*W	3	0.000050	0.000017	30.2	0.000
D*R*W	3	0.000013	0.000004	7.5	0.000
Error	32	0.000018	0.000001		
General	47	0.001979			
R, (m^2^·K)/W					
Absolute term	1	399.1361	399.1361	117,304.8	0.000000
D	1	0.7508	0.7508	220.7	0.000000
R	3	3.1676	1.0559	310.3	0.000000
W	1	7.7174	7.7174	2268.1	0.000000
D*R	3	0.0386	0.0129	3.8	0.019845
D*W	1	0.0183	0.0183	5.4	0.026964
R*W	3	0.0785	0.0262	7.7	0.000525
D*R*W	3	0.0323	0.0108	3.2	0.037636
Error	32	0.1089	0.0034		
General	47	11.9125			

## Data Availability

The original contributions presented in this study are included in the article. Further inquiries can be directed to the corresponding author.
